# Unraveling the prognostic significance of RGS gene family in gastric cancer and the potential implication of RGS4 in regulating tumor-infiltrating fibroblast

**DOI:** 10.3389/fmolb.2024.1158852

**Published:** 2024-04-17

**Authors:** Yalan Yang, Siyuan Xing, Xi Luo, Lulu Guan, Yao Lu, Yiting Wang, Feng Wang

**Affiliations:** Department of Oncology, First Affiliated Hospital of Zhengzhou University, Zhengzhou, China

**Keywords:** RGS4, gastric cancer, prognosis, fibroblast, immune infiltration

## Abstract

Regulator of G-protein signaling (RGS) proteins are regulators of signal transduction mediated by G protein-coupled receptors (GPCRs). Current studies have shown that some molecules in the RGS gene family are related to the occurrence, development and poor prognosis of malignant tumors. However, the RGS gene family has been rarely studied in gastric cancer. In this study, we explored the mutation and expression profile of RGS gene family in gastric cancer, and evaluated the prognostic value of RGS expression. Then we established a prognostic model based on RGS gene family and performed functional analysis. Further studies showed that RGS4, as an independent prognostic predictor, may play an important role in regulating fibroblasts in the immune microenvironment. In conclusion, this study explores the value of RGS gene family in gastric cancer, which is of great significance for predicting the prognosis and guiding the treatment of gastric cancer.

## 1 Introduction

Gastric cancer is one of the most common malignant tumors of the digestive tract worldwide, with high incidence rate of 5.6% and cancer-related mortality rate of 7.7% (Suzuki et al., 2016; Sung et al., 2021). In spite of improvements in the clinical treatment strategies (Smyth et al., 2020), patients with gastric cancer still face a dire survival situation due to the high heterogeneity of tumor cells (Jiang et al., 2022) and the complex tumor microenvironment (TME) composed of stromal and immune cells (Kumar et al., 2022). As a result of gastric cancer’s high heterogeneity, it is important to explore precise and individualized predictive biomarkers at the molecular level for the clinical precision treatment and prognostic monitoring of gastric cancer.

The current drug targets of extremely interest are G protein-coupled receptors (GPCRs) (Nieto Gutierrez and McDonald, 2018; Sriram and Insel, 2018), which play key roles in the regulation of cell homeostasis, cell signal transduction, immune system and nervous system (Calebiro et al., 2021; Nagai et al., 2021; Cheng et al., 2022). There is increasing evidence that the expression and activation of GPCR family proteins are involved in the development of numerous types of tumors (Chaudhary and Kim, 2021). The regulator of G-protein signaling (RGS) is a diverse family of functional proteins, share a domain with a conserved core that includes 120 amino acid residues, that accelerate the deactivation of heterotrimeric G-protein and modulate signaling initiated by GPCRs (Hurst et al., 2009; Hurst and Hooks, 2009; Guda et al., 2020; Li C. et al., 2021). Previous studies have demonstrated that many molecules in RGS gene family are associated with the occurrence, development and prognosis of malignant tumors (Hurst and Hooks, 2009; Guda et al., 2020; Yang et al., 2023). At present, the studies on RGS gene family in gastric cancer are rare and the pathogenic mechanism has not been fully investigated. Its regulation on the proliferation, metabolism, immune regulation and prognosis of gastric cancer has been preliminarily studied (Wang et al., 2017; Li W. et al., 2020; Yang et al., 2022). High expression of RGS1 and RGS3 is associated with poor prognosis in patients with gastric cancer (Wang et al., 2017; Li S. et al., 2021). RGS2 deposition in gastric cancer is associated with increased tumor stage (Yang et al., 2022). The expression of RGS5 was negatively correlated with microvascular density, which may be related to abnormal formation of blood vessels (Wang et al., 2010). Our work comprehensively explored important role of RGS gene family in gastric cancer for the first time and speculated that the RGS gene family, particularly RGS4, could be a prognostic and therapeutic target for gastric cancer.

In this study, we aimed to comprehensively evaluate the mutation and expression profiles of RGS gene family and explore the relationship between the expression and survival outcomes for patients with gastric cancer. A risk score model was constructed based on RGS gene family data to predict the survival of patients with gastric cancer. In addition, we investigated the function of RGS gene family, especially RGS4, in regulating gastric cancer formation and tumor microenvironment.

## 2 Materials and methods

### 2.1 Data processing

GSE66229, GSE13861 and GSE84433 were downloaded from the Gene Expression Omnibus (GEO) database (https://www.ncbi.nlm.nih.gov). The transcriptome and somatic mutation data of stomach adenocarcinoma (STAD) were downloaded from TCGA database (https://portal.gdc.cancer.gov). Tumor Immune Single-cell Hub (TISCH, http://tisch.comp-genomics.org) was used to analyze the single cell sequencing dataset GSE167297 (Sun et al., 2021). The drug data were downloaded from CellMiner database (https://discover.nci.nih.gov/cellminer/home.do) (Reinhold et al., 2012).

### 2.2 Somatic mutations and copy number alterations of RGS family

Summary analysis of somatic mutation frequency in the 22 RGS genes was performed using cBioPortal for Cancer Genomics (https://www.cbioportal.org) (Cerami et al., 2012). Additionally, we calculated the percentage of the population with increased and missing somatic copy numbers of RGS family genes in the TCGA-STAD cohort and plotted them using the R language function “barplot”. The “RCircos” package was further used to visualize the chromosomal locations of family genes (Zhang et al., 2013).

### 2.3 Survival analysis of RGS genes and construction of a prognostic signature

The box diagram showed the expression of RGS family genes in normal and tumor tissues by the “ggboxplot” R software package. Kaplan-Meier analysis was used to evaluate the prognostic value of RGS family and the forest map was drawn using the “forestplot” R software package, of which a *p*-value of less than 0.05 were considered the genes that significantly impact the survival for patients with gastric cancer. A risk prognosis model composed of 3 genes was established based on the multivariate Cox regression analyses of training set GSE66229. The median value of risk score was used to separate samples into high- and low-risk groups in the training set and the other three test cohorts. Survival curves were drawn by the “survival” and “survminer” R packages.

### 2.4 DEG identification and functional analysis

Differentially expressed genes (DEG) were selected between the different risk groups using wilcox test with a *p*-value<0.05. To identify the functions and biological processes of each subgroup, gene set variation analysis (GSVA) was performed, which was based on the hallmark gene set downloaded from the MSigDB database (Hänzelmann et al., 2013; Liberzon et al., 2015). Kyoto Encyclopedia of Genes and Genomes (KEGG) and Gene Ontology (GO) pathway analysis was performed by R software to assess the potential functions of genes. The “clusterprofiler” R package was used for GSEA (https://www.gsea-msigdb.org/gsea/index.jsp) (Subramanian et al., 2005; Yu et al., 2012).

### 2.5 Immune landscape

The Stromal and Immune Scores were calculated using the “estimate” R package (Yoshihara et al., 2013). Higher ESTIMATE Scores correspond to lower tumor purity. Tumor-infiltrating immune cells across cancers were analyzed using the “MCPcounter” R package (Becht et al., 2016).

### 2.6 Correlation analysis and intersection gene acquisition

Spearman correlation analysis was implemented to analyze the correlation between RGS4 and other continuity variables by using “cor.test” function. The intersection genes of the RGS4-related genes in the training and validation sets were obtained by the “venn” R package. And the filtering criterion of correlation coefficient is 0.4. In addition, we use “ggplot” function to plot correlation scatter plots.

### 2.7 Statistical analyses

R 4.2.0 software (https://www.R-project.org) and Adobe Photoshop CS6 were used for statistical analysis and graphing in this article. Statistical significance was considered *p* < 0.05, and all *p*-values were two tailed.

## 3 Results

### 3.1 Mutation landscape and expression analysis of RGS gene family in gastric cancer

**FIGURE 1:**
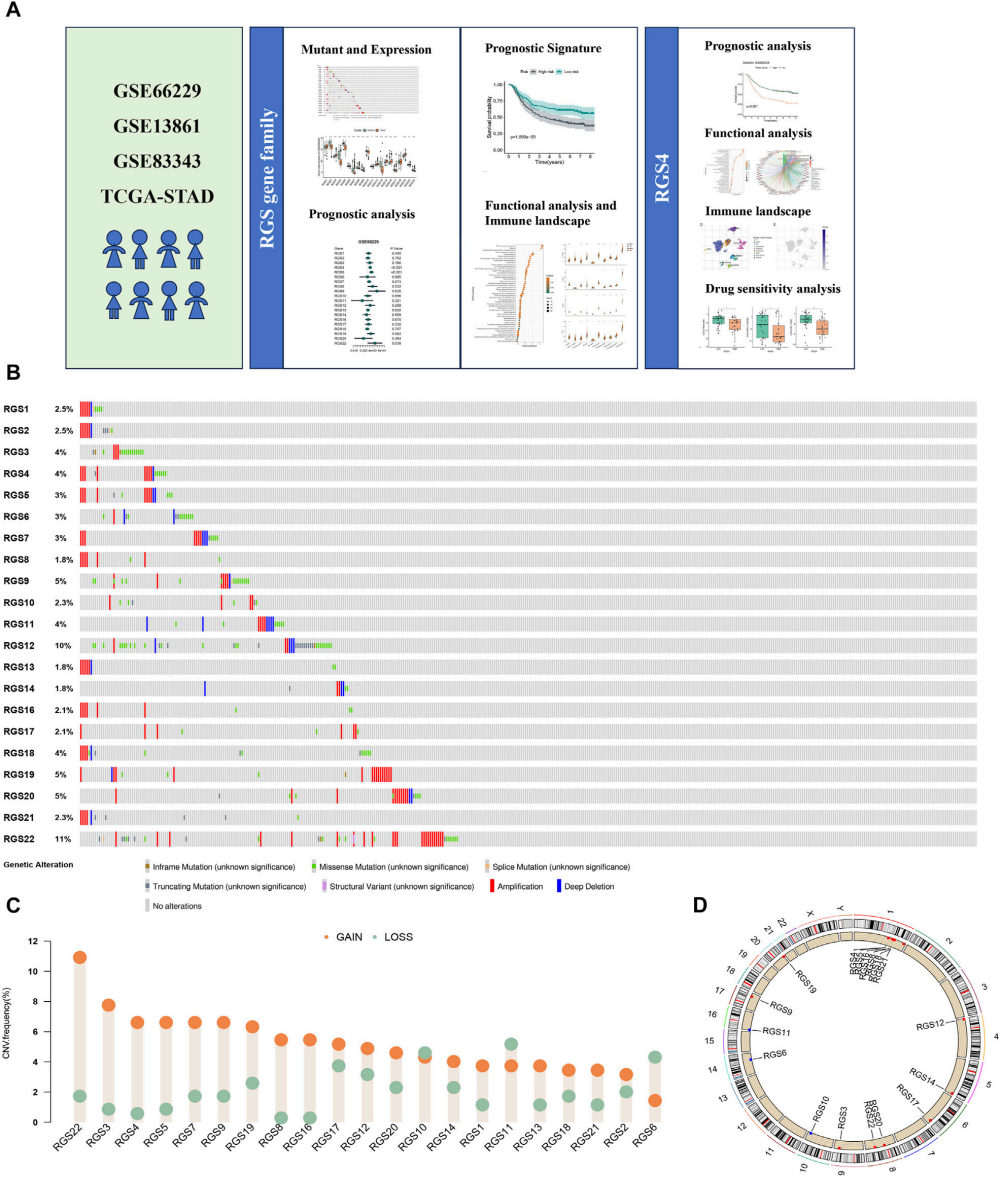
Somatic Mutations and Copy Number Alterations of RGS Family in gastric cancer. **(A)** The graphical abstract. **(B)**Type and frequency of mutations. **(C)** The copy number increase or decrease ratio of RGS family genes in patients with gastric cancer in STAD. **(D)** The location of RGS family genes on different chromosomes.

A total of 21 RGS genes were included for mutations analysis, showing different mutation frequencies and types, among which the main mutation type was amplification (Figure 1B). Among RGS gene family, RGS22 had relatively higher mutational frequencies of 11%. Different CNV frequencies of all RGS genes were showed by the assessment of copy number variations (CNV) (Figure 1C). RGS22, RGS3, RGS4 and RGS5 exhibited a significant increase of copy number gain. Figure 1D shows the locations of all 21 RGS genes in different chromosomes.

**FIGURE 2:**
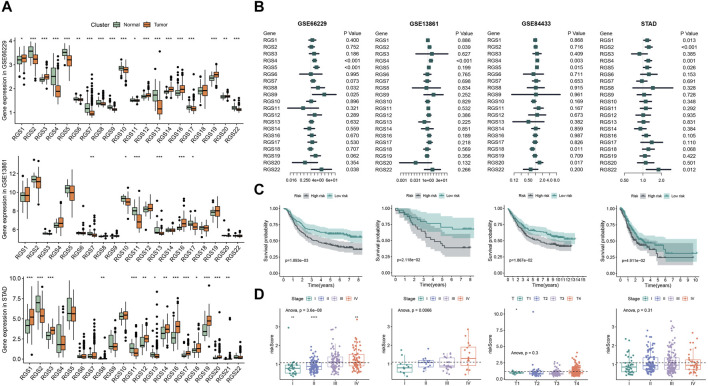
Prognostic analysis of RGS genes expression in gastric cancer. **(A)** The expression of RGS gene family of normal tissue and gastric cancer in GSE66229, GSE13861 and STAD. **(B)** Cox analysis of RGS gene family in GSE66229, GSE13861, GSE84433 and STAD. **(C)** Kaplan-Meier survival analysis of prognostic features constructed by RGS genes. **(D)** Risk scores for patients at different stages. *p < 0.05, **p < 0.01, ***p < 0.001, ****p < 0.0001.

Then we analyzed the mRNA expression of RGS gene family of gastric cancer from the GEO and STAD databases (Figure 2A). The box map showed that the expression of RGS1, RGS3, RGS12, RGS14, RGS16, RGS19, RGS20 was significantly upregulated in the gastric tissues, while the expression of RGS2, RGS4, RGS5, RGS6, RGS7, RGS8, RGS9, RGS10, RGS13, RGS17 was significantly lower.

### 3.2 Prognostic value of RGS genes in gastric cancer

To uncover the association between the expression of RGS family genes and prognosis, we performed Cox survival analysis. The univariate Cox analysis showed that the expression of RGS4, RGS5, RGS8, RGS9 and RGS22 was associated with poor prognosis in the train set GSE66229 (Figure 2B). Subsequent multivariate Cox regression was performed and 3 prognostic RGS genes was used to construct prognostic features, including RGS4, RGS5 and RGS22. Kaplan-Meier survival analysis revealed that patients with high risk had poorer prognosis in both the train set and three test sets (Figure 2C). Further, the risk score was significantly different between different tumor stages for patients in GSE66229 and GSE13861 (Figure 2D).

### 3.3 Pathway enrichment analysis

**FIGURE 3:**
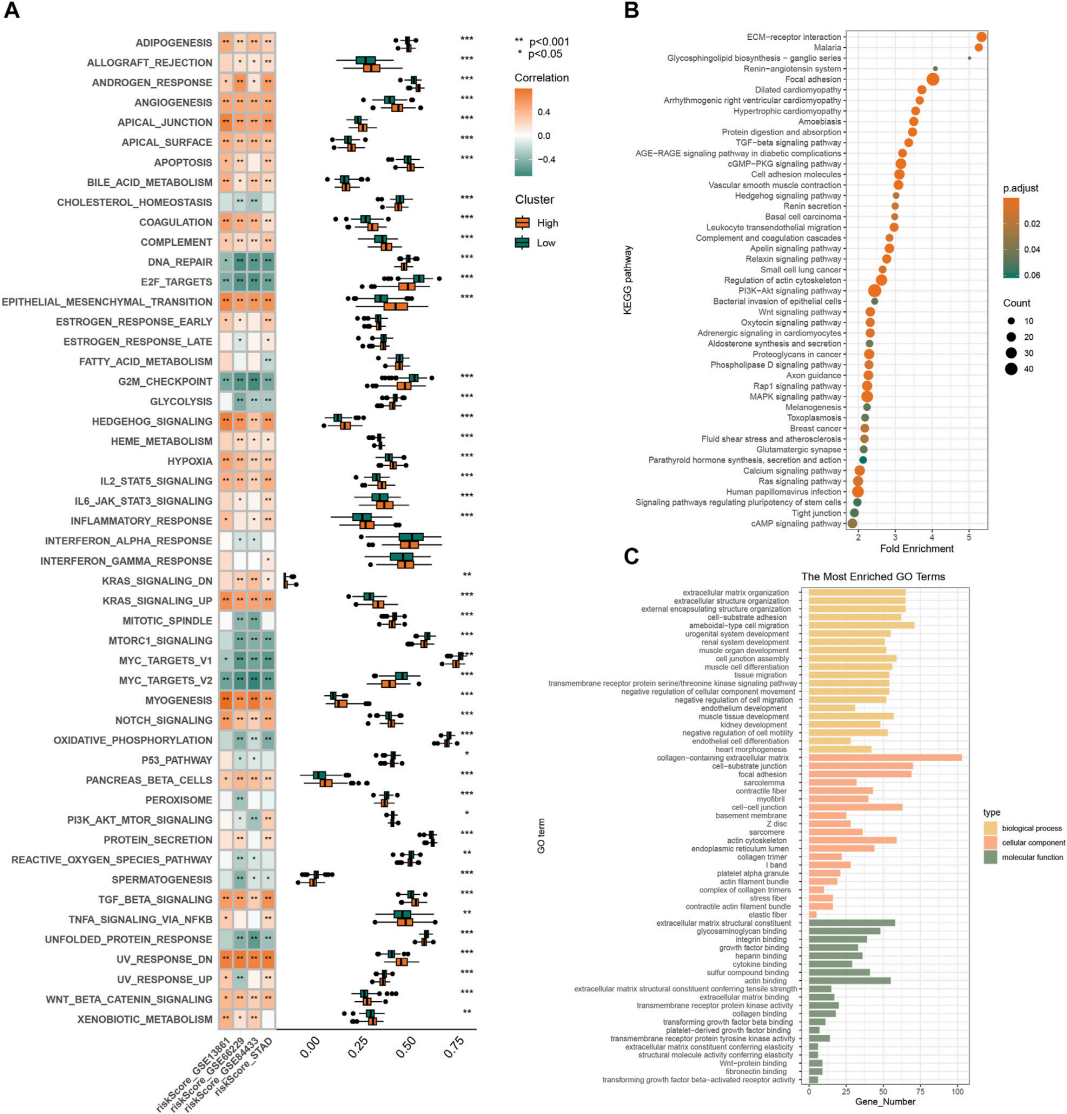
Gene enrichment analysis by bioinformatics analysis. **(A)** Pathway enrichment and correlation analysis of prognostic features. KEGG **(B)** and GO **(C)** analyses of differential genes in high and low risk groups in GSE66229.

Based on the prognostic risk model made up of three RGS genes, we further explored possible mechanisms to explain the higher risk of death and poorer clinical stage in the high-risk subgroup. Figure 3A shows that pathways such as angiogenesis, hypoxia, apical junction, epithelial mesenchymal transformation, and myogenesis, which are involved in matrix remodeling of tumor microenvironment and promote tumor cell metastasis, are highly enriched in the high-risk subgroup. This is consistent with the tendency of high-risk populations to have worse clinical staging as shown in Figure 2D. In addition, signaling pathways closely related to tumor cell proliferation and differentiation, such as upregulation of KRAS signaling, dysregulation of hedgehog signaling, NOTCH signaling and TGFβ signaling, were also highly enriched in the high-risk subgroup. The pathway analysis of KEGG (Figure 3B) and GO (Figure 3C) confirmed above results. The results of functional analysis suggest that the prognostic signal constructed by RSG family genes may play an important role in the occurrence, development and metastasis of tumors, and the patients with high scores have worse clinical stage and shorter survival time.

### 3.4 Correlation between risk score and immune infiltration

**FIGURE 4:**
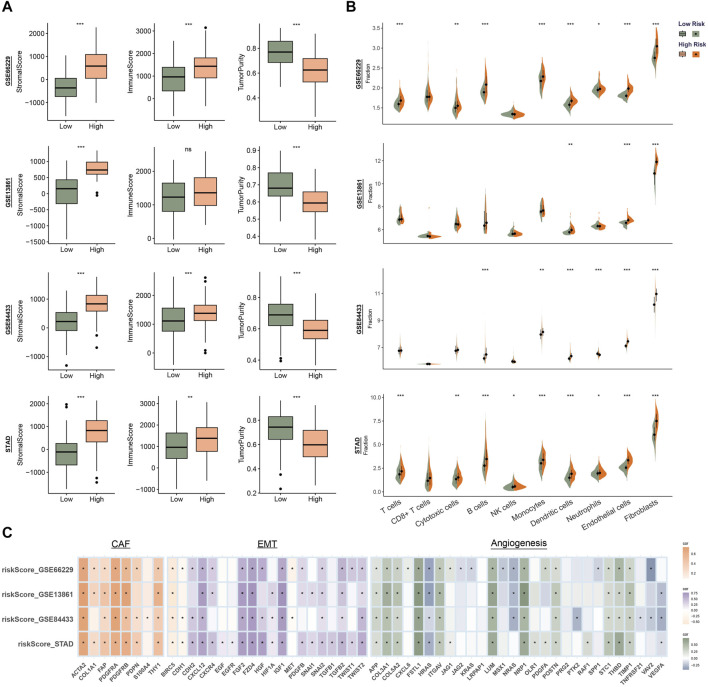
Identify the immune microenvironment landscape associated with prognostic features in GSE66229, GSE13861, GSE84433 and STAD. **(A)** Evaluation of the ESTIMATE Scores. **(B)** Quantity of immunological infiltration cells as determined by MCPcounter. **(C)** Correlation heatmap of risk score with fibroblast, EMT and angiogenesis associated genes. *p < 0.05, **p < 0.01, ***p < 0.001.

TME is a complex system consisting of immune microenvironment dominated by immune cells and non-immune microenvironment dominated by fibroblasts. To evaluate the correlation between risk score and features of the TME, we calculated the immune scores, stromal scores and tumor purity. As shown in Figure 4A, patients with high risk had higher stromal score and lower tumor purity. We evaluate the infiltration of different types of immune cells and found that fibroblasts and endothelial cells were higher in patients of high-risk group in all four sets (Figure 4B). We hypothesized that the RGS family genes might be able to reshape a stromal cell-rich immune microenvironment. Further analysis also showed that the risk score was associated with genes in the fibroblast, epithelial interstitial transformation, and angiogenesis pathways (Figure 4C and Supplementary Figure S1).

### 3.5 Analysis of the relationship between RGS4 expression and prognosis in GC

**FIGURE 5:**
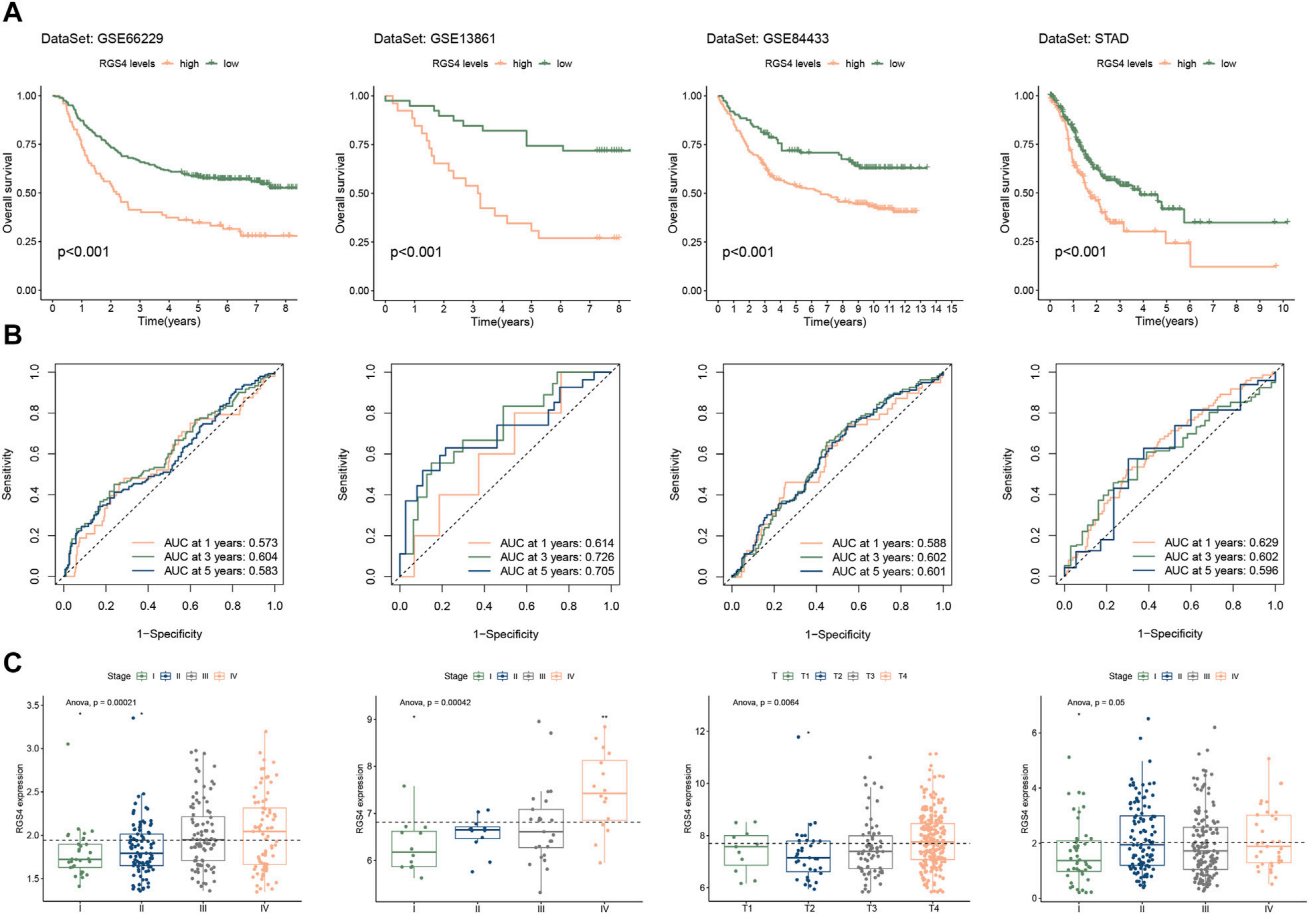
Evaluation of prognostic value and clinical features of RGS4 expression. Kaplan-Meier survival analysis **(A)** and time-dependent ROC curve **(B)** of RGS4 expression in GSE66229, GSE13861, GSE84433 and STAD. **(C)** Expression of RGS4 in patients with different clinical stages or depth of invasion.

Cox analysis showed that the expression of RGS4 was associated with poor prognosis in all four sets (Figure 2B). However, for RGS5 and RGS22 in part of the validation cohort, univariate cox analysis results did not meet the statistical difference. We further analyzed the effect of RGS4 on prognosis in all gastric cancer cohorts. Kaplan-Meier survival analysis and time-dependent ROC curves of the expression of RGS4 were plotted in Figure 5. Further analysis found that RGS4 expression was closely associated with tumor stage, increased from stage I to other stages (Figure 5C).

### 3.6 Role of RGS4 in remodeling CAF-enriched tumor microenvironments

**FIGURE 6:**
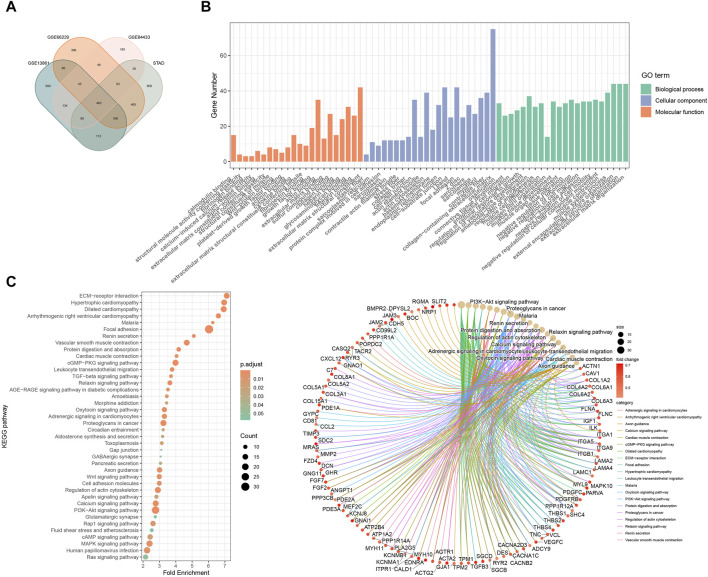
Functional enrichment analysis of RGS4-related genes. **(A)** Veen plot of the intersection of RGS-related genes. GO **(B)** and KEGG **(C)** analyses of RGS4-related genes.

**FIGURE 7:**
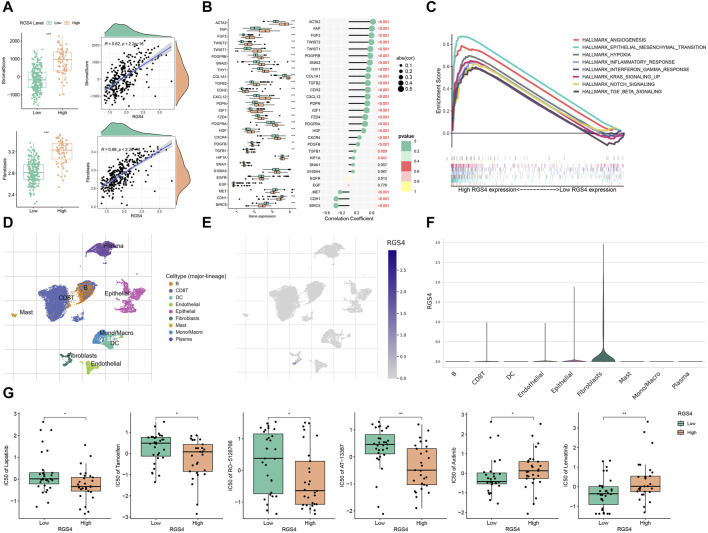
Modulation of RGS4 on tumor microenvironment. **(A)** The stromal score and the infiltration of fibroblasts of the RGS4 high- and low-group in GSE66229. **(B)** Evaluation of the expression of fibroblast and EMT-related genes in different RGS4 expression groups in GSE66229. **(C)** GSEA analysis in different RGS4 expression groups. **(D)** Distribution of different immune cells in GSE167297. **(E)** Expression distribution of RGS4 in different immune cells in GSE167297. **(F)** Violin diagram of RGS4 expression distribution in different immune cells of GSE167297. **(G)** Drug susceptibility analysis. *p < 0.05, **p < 0.01, ***p < 0.001.

Function analysis was performed and showed that RGS4-related genes were enriched in matrix formation-related pathways, which was consistent with the above results (Figure 6 and Supplementary Figure S2). Further analysis showed that RGS4 expression was positively correlated with matrix score and fibroblast expression (Figure 7A and Supplementary Figure S3). The expression of genes related to fibroblast and epithelial interstitial transformation was highly consistent with that of RGS4 (Figure 7B). The GSEA analysis showed that patients with high RGS4 expression were highly enriched in pathways related to angiogenesis and EMT (Figure 7C). Single cell sequencing dataset GSE167297 confirmed that RGS4 was highly expressed in fibroblasts (Figures 7D–F).

### 3.7 Drug sensitivity analysis

The drugs related to the RGS4 were downloaded from the CellMiner database. The results showed that patients with high RGS4 expression were more sensitive to Lapatinib, Tamoxifen, RO-5126766 and AT-13387, while patients with low RGS4 expression were more likely to benefit from Axitinib and Lenvatinib (Figure 7G).

## 4 Discussion

Recently, more and more studies have shown that RGS family genes, as regulators of GPCRs, play an important role in the occurrence, development and prognosis of many cancers and has been proved to be potential drug targets for the treatment of malignant tumors (Hurst and Hooks, 2009; Dasgupta et al., 2021; Weisshaar et al., 2022; Zhang et al., 2022). Here, we systematically analyzed the role of RGS family genes in the tumor microenvironment and the prognostic value of gastric cancer for the first time. The results showed that amplification and missense mutations of RGS family genes are common in gastric cancer. Most RGS genes, especially RGS22, RGS3, RGS4, and RGS5, have copy number amplification. Previous studies have shown that gene mutations and abnormal DNA copy number changes are important molecular mechanisms of many human diseases (Tang and Amon, 2013; Martincorena and Campbell, 2015; Hollox et al., 2022). Genome-wide CNV is often used for disease detection, visualizing the deletion or amplification of genomic DNA from tumors and hereditary diseases (Panda et al., 2020). For tumors, the missing fragment may contain tumor suppressor genes, while the amplified fragment may contain oncogenes. The amplified RGS family genes may be closely related to the pathogenesis of cancer, which arouses our interest to further explore the role of RGS family genes in the occurrence and development of gastric cancer.

Further studies confirmed that some RGS family genes are associated with poor prognosis of gastric cancer. High expression of RGS1, RGS2 and RGS3 in patients with gastric cancer has been shown to be associated with poor prognosis or poor tumor stage (Wang et al., 2017; Li S. et al., 2021; Yang et al., 2022). Our results suggest that RGS1 and RGS2 are adverse prognostic factors in some gastric cancer cohorts. RGS3 does not appear to be associated with prognosis in patients with gastric cancer, whereas RGS5, which has been shown to be associated with abnormal vascular formation, is a prognostic risk factor in three gastric cancer cohorts (Wang et al., 2010). We further constructed a 3 RGS genes-related prognostic signature to predict the prognosis of patients with gastric cancer, and found that the high-risk subgroup having a worse prognosis and higher tumor stage.

The occurrence, development and prognosis of tumors are closely related to the activation of tumor signals and the remodeling of the surrounding microenvironment. In the high-risk group, a variety of pathways involved in remodeling tumor microenvironment matrix and promoting tumor metastasis (such as angiogenesis, hypoxia, apical junction, epithelial mesenchymal transformation, myogenesis, *etc.*) and pathways closely related to tumor cell proliferation and differentiation (upregulation of KRAS signal, dysfunction of hedgehog signal, NOTCH signal and TGFβ signal, *etc.*) were highly enriched. These pathways contribute greatly to tumor genesis, progression and metastasis (Shimo, 2020; Song and Zhou, 2021; Xing et al., 2021; Qing et al., 2022). At the same time, the results of tumor microenvironment analysis showed that the high-risk group had higher interstitial scores and fibroblast infiltration. It is well known that the tumor microenvironment is composed of tumor cells and their surrounding immune and matrix components, which play different roles in the development of tumors (Xiao and Yu, 2021). The interaction of non-neoplastic stromal cells, particularly cancer-associated fibroblasts, with tumor cells contributes to the formation and spread of malignant solid tumors (Huang et al., 2022; Jenkins et al., 2022). Our results suggest that the RGS family may be involved in the regulation of the stromal components of the tumor microenvironment.

Considering that RGS4 is associated with poor prognosis in four gastric cancer data sets, we then focused on exploring the effect of RGS4 on the prognosis and treatment of gastric cancer. At present, RGS4 family proteins as a new regulatory factor of malignant tumors, its role has not been well proved. RGS4 has recently been studied as a tumor promoter in glioblastoma (Bao et al., 2020; Guda et al., 2020), non-small cell lung cancer (He et al., 2019) and osteosarcoma tumors (Liu et al., 2020), and is reported to be a potent driver of cell proliferation, invasion and migration. Studies have shown that overexpression of RGS4 is associated with the development and poor prognosis of glioblastoma (Bao et al., 2020) and non-small cell lung cancer (He et al., 2019). However, some studies have reported that increased RGS4 protein significantly inhibits cell migration and invasion in breast cancer (Xie et al., 2009) and loss of RGS4 is associated with poor prognosis in pediatric nephroblastoma (Liu et al., 2017). Our study found that RGS4 is closely related to signaling pathways related to tumor formation and metastasis.

Here, for the first time, we studied the correlation between RGS4 and fibroblasts, and found that RGS4 was significantly positively correlated with fibroblast infiltration in the tumor immune microenvironment. The expression of RGS4 was significantly positively correlated with the expression of CAFs biomarkers (ACTA2, FAP, PDGFRB) and EMT markers (FGF2, Twist2, Twist1). Recent studies have shown that gastric cancer patients with high expression of ACTA2 have poor prognosis and poor response to immunotherapy (Park et al., 2023). Previous studies have shown that the main function of FGF2 is related to cell adhesion and angiogenesis, and patients with high FGF2 expression level have poor TNM stage and prognosis (Li Y. et al., 2020). Zhao et al. found that Twist1 was an independent factor affecting the pathological response to neoadjuvant chemotherapy for gastric cancer, and the expression of FAP in CAF was a significant factor for poor prognosis in patients with gastric cancer (Tong et al., 2022; Zhao and Zhu, 2023). The expression of PDGFB has been reported to be closely related to tumor metastasis in patients with gastric cancer (Han et al., 2020; Du et al., 2022). In addition, single-cell sequencing data further confirmed the high expression of RGS4 in fibroblasts. Our results suggest that high expression of RGS4 is closely related to fibroblast infiltration and stroma formation in the tumor immune microenvironment, which may promote tumor development and metastasis.

In addition, there are some limitations in this study. There may be population selection bias based on public datasets. Further cell and animal experiments are needed to further investigate the molecular mechanism of RGS gene family regulation. In conclusion, our study provides a new perspective for exploring the mechanism of RGS family genes in the development of gastric cancer and proposes possible therapy through drug sensitivity studies, which is critical for precision medicine in gastric cancer.

## Data Availability

Publicly available datasets were analyzed in this study. This data can be found here: TCGA (https://portal.gdc.cancer.gov) and GEO (https://www.ncbi.nlm.nih.gov) databases. Single cell sequencing dataset was analyzed by Tumor Immune Single-cell Hub (TISCH, http://tisch.comp-genomics.org/).
