# Supplementary material for: Unraveling the prognostic significance of RGS gene family in gastric cancer and the potential implication of RGS4 in regulating tumor-infiltrating fibroblast
Source: Front Mol Biosci. 2024 Apr 17;11:1158852. doi: 10.3389/fmolb.2024.1158852 (PMC11061405; doi:10.3389/fmolb.2024.1158852)
Supplement: Supplementary file 1 [file Table1.docx]

Supplementary Tables

**Supplementary Table 1** Univariate cox analysis of RGS gene family in GSE66229.

| **Gene** | **HR** | **HR.95L** | **HR.95H** | ***P* value** |
| --- | --- | --- | --- | --- |
| RGS1 | 1.3432767 | 0.67544239 | 2.671422785 | 0.400167237 |
| RGS2 | 1.1007551 | 0.60633189 | 1.998347591 | 0.752370266 |
| RGS3 | 1.8996941 | 0.73474271 | 4.911702607 | 0.185506349 |
| RGS4 | 2.0789644 | 1.42118958 | 3.041179731 | 0.000162523 |
| RGS5 | 3.0425426 | 1.7722008 | 5.223485526 | 5.46E-05 |
| RGS6 | 0.9932249 | 0.10784885 | 9.147020187 | 0.995211695 |
| RGS7 | 1.5581728 | 0.95964738 | 2.529994348 | 0.072907534 |
| RGS8 | 4.1282565 | 1.12597498 | 15.13577282 | 0.032439176 |
| RGS9 | 14.505852 | 1.39484179 | 150.8556376 | 0.02518927 |
| RGS10 | 1.0616411 | 0.43341111 | 2.600491275 | 0.895884644 |
| RGS11 | 0.2114658 | 0.00982428 | 4.551761154 | 0.321113642 |
| RGS12 | 2.1054898 | 0.53151958 | 8.34040243 | 0.289101533 |
| RGS13 | 1.1158641 | 0.71281691 | 1.746805757 | 0.631620357 |
| RGS14 | 0.73558 | 0.2625996 | 2.060467285 | 0.558984992 |
| RGS16 | 1.1760905 | 0.55720315 | 2.4823781 | 0.670432762 |
| RGS17 | 1.3180265 | 0.55669844 | 3.120529582 | 0.53003192 |
| RGS18 | 0.9080804 | 0.549067 | 1.501838704 | 0.707191777 |
| RGS19 | 2.5345092 | 0.95308004 | 6.739976431 | 0.062369188 |
| RGS20 | 0.3067984 | 0.02515798 | 3.741368825 | 0.354470329 |
| RGS22 | 8.2492269 | 1.12086854 | 60.7116194 | 0.038264795 |

**Supplementary Table 2** Univariate cox analysis of RGS gene family in GSE13861.

| **Gene** | **HR** | **HR.95L** | **HR.95H** | ***P* value** |
| --- | --- | --- | --- | --- |
| RGS1 | 0.976086 | 0.701754 | 1.357659 | 0.885678 |
| RGS2 | 1.493978 | 1.021267 | 2.185492 | 0.038603 |
| RGS3 | 2.716646 | 0.048436 | 152.3706 | 0.626667 |
| RGS4 | 2.132714 | 1.419939 | 3.203283 | 0.000263 |
| RGS5 | 1.25674 | 0.886593 | 1.781421 | 0.199224 |
| RGS6 | 0.871267 | 0.353284 | 2.148716 | 0.764775 |
| RGS7 | 0.850778 | 0.376079 | 1.924655 | 0.698021 |
| RGS8 | 0.686067 | 0.020077 | 23.44437 | 0.834357 |
| RGS9 | 4.1108 | 0.366905 | 46.05737 | 0.251522 |
| RGS10 | 0.941919 | 0.547512 | 1.620441 | 0.82886 |
| RGS11 | 1.107644 | 0.803634 | 1.526661 | 0.532281 |
| RGS12 | 1.270898 | 0.739223 | 2.184971 | 0.385901 |
| RGS13 | 0.412939 | 0.098975 | 1.722843 | 0.224912 |
| RGS14 | 0.868668 | 0.199176 | 3.788525 | 0.851372 |
| RGS16 | 1.376679 | 0.85474 | 2.217335 | 0.188666 |
| RGS17 | 1.515385 | 0.78167 | 2.937804 | 0.218444 |
| RGS18 | 0.787371 | 0.345703 | 1.793311 | 0.569203 |
| RGS19 | 0.805421 | 0.50891 | 1.274692 | 0.355586 |
| RGS20 | 0.044919 | 0.00079 | 2.554091 | 0.132294 |
| RGS22 | 3.138379 | 0.419119 | 23.50029 | 0.265535 |

**Supplementary Table 3** Univariate cox analysis of RGS gene family in GSE13861.

| **Gene** | **HR** | **HR.95L** | **HR.95H** | ***P* value** |
| --- | --- | --- | --- | --- |
| RGS1 | 0.987468 | 0.851036 | 1.145771 | 0.867969 |
| RGS2 | 1.029353 | 0.880705 | 1.203091 | 0.716184 |
| RGS3 | 1.181732 | 0.795154 | 1.75625 | 0.408783 |
| RGS4 | 1.215423 | 1.068994 | 1.38191 | 0.002896 |
| RGS5 | 1.170817 | 1.031724 | 1.328661 | 0.014526 |
| RGS6 | 0.832361 | 0.31571 | 2.194498 | 0.710662 |
| RGS7 | 1.08763 | 0.754504 | 1.567837 | 0.65256 |
| RGS8 | 1.045839 | 0.459502 | 2.380357 | 0.914938 |
| RGS9 | 1.060282 | 0.100464 | 11.19 | 0.96117 |
| RGS10 | 0.856484 | 0.68693 | 1.067889 | 0.168701 |
| RGS11 | 1.376294 | 0.875041 | 2.164679 | 0.166888 |
| RGS12 | 1.196098 | 0.52085 | 2.746763 | 0.672913 |
| RGS13 | 0.509093 | 0.112006 | 2.31395 | 0.382149 |
| RGS14 | 1.0354 | 0.705897 | 1.518712 | 0.858732 |
| RGS16 | 0.998306 | 0.811233 | 1.228519 | 0.987224 |
| RGS17 | 0.975323 | 0.780112 | 1.219383 | 0.826431 |
| RGS18 | 0.750562 | 0.60118 | 0.937064 | 0.011275 |
| RGS19 | 0.962505 | 0.787257 | 1.176765 | 0.709392 |
| RGS20 | 1.497067 | 1.074605 | 2.085611 | 0.017065 |
| RGS22 | 0.632511 | 0.313878 | 1.274604 | 0.2001 |

**Supplementary Table 4** Univariate cox analysis of RGS gene family in STAD.

| **Gene** | **HR** | **HR.95L** | **HR.95H** | ***P* value** |
| --- | --- | --- | --- | --- |
| RGS1 | 1.17383 | 1.034686 | 1.331684 | 0.012787 |
| RGS2 | 1.243939 | 1.093106 | 1.415584 | 0.000934 |
| RGS3 | 0.878438 | 0.655566 | 1.177079 | 0.385368 |
| RGS4 | 1.223291 | 1.080315 | 1.38519 | 0.001482 |
| RGS5 | 1.173161 | 1.018825 | 1.350876 | 0.026479 |
| RGS6 | 1.250532 | 0.92001 | 1.699798 | 0.153407 |
| RGS7 | 0.961636 | 0.793008 | 1.166123 | 0.690875 |
| RGS8 | 1.450484 | 0.688364 | 3.056383 | 0.328096 |
| RGS9 | 1.04706 | 0.808302 | 1.356342 | 0.727648 |
| RGS10 | 1.108911 | 0.893667 | 1.375996 | 0.347775 |
| RGS11 | 1.116386 | 0.909608 | 1.370171 | 0.292139 |
| RGS12 | 1.01135 | 0.771279 | 1.326148 | 0.93494 |
| RGS13 | 1.022965 | 0.829972 | 1.260836 | 0.831437 |
| RGS14 | 0.909007 | 0.733159 | 1.127032 | 0.384443 |
| RGS16 | 1.13141 | 0.974713 | 1.313299 | 0.104541 |
| RGS17 | 1.390455 | 0.927618 | 2.084226 | 0.110457 |
| RGS18 | 1.227586 | 0.985128 | 1.529717 | 0.067776 |
| RGS19 | 1.079381 | 0.89571 | 1.300714 | 0.42217 |
| RGS20 | 1.110384 | 0.818263 | 1.506794 | 0.501431 |
| RGS22 | 1.789603 | 1.134722 | 2.822436 | 0.012291 |

**Supplementary Table 5** Multivariate cox analysis of RGS gene family in STAD.

| **Gene** | **coef** | **HR** | **HR.95L** | **HR.95H** | ***P* value** |
| --- | --- | --- | --- | --- | --- |
| RGS4 | 0.167243 | 1.182041 | 1.009488 | 1.384089 | 0.037779 |
| RGS5 | 0.281676 | 1.325349 | 1.107076 | 1.586656 | 0.002156 |
| RGS22 | 0.114799 | 1.121648 | 0.960945 | 1.309226 | 0.145659 |
