# Supplementary figures and images for: Unraveling the prognostic significance of RGS gene family in gastric cancer and the potential implication of RGS4 in regulating tumor-infiltrating fibroblast
Source: Front Mol Biosci. 2024 Apr 17;11:1158852. doi: 10.3389/fmolb.2024.1158852 (PMC11061405; doi:10.3389/fmolb.2024.1158852)

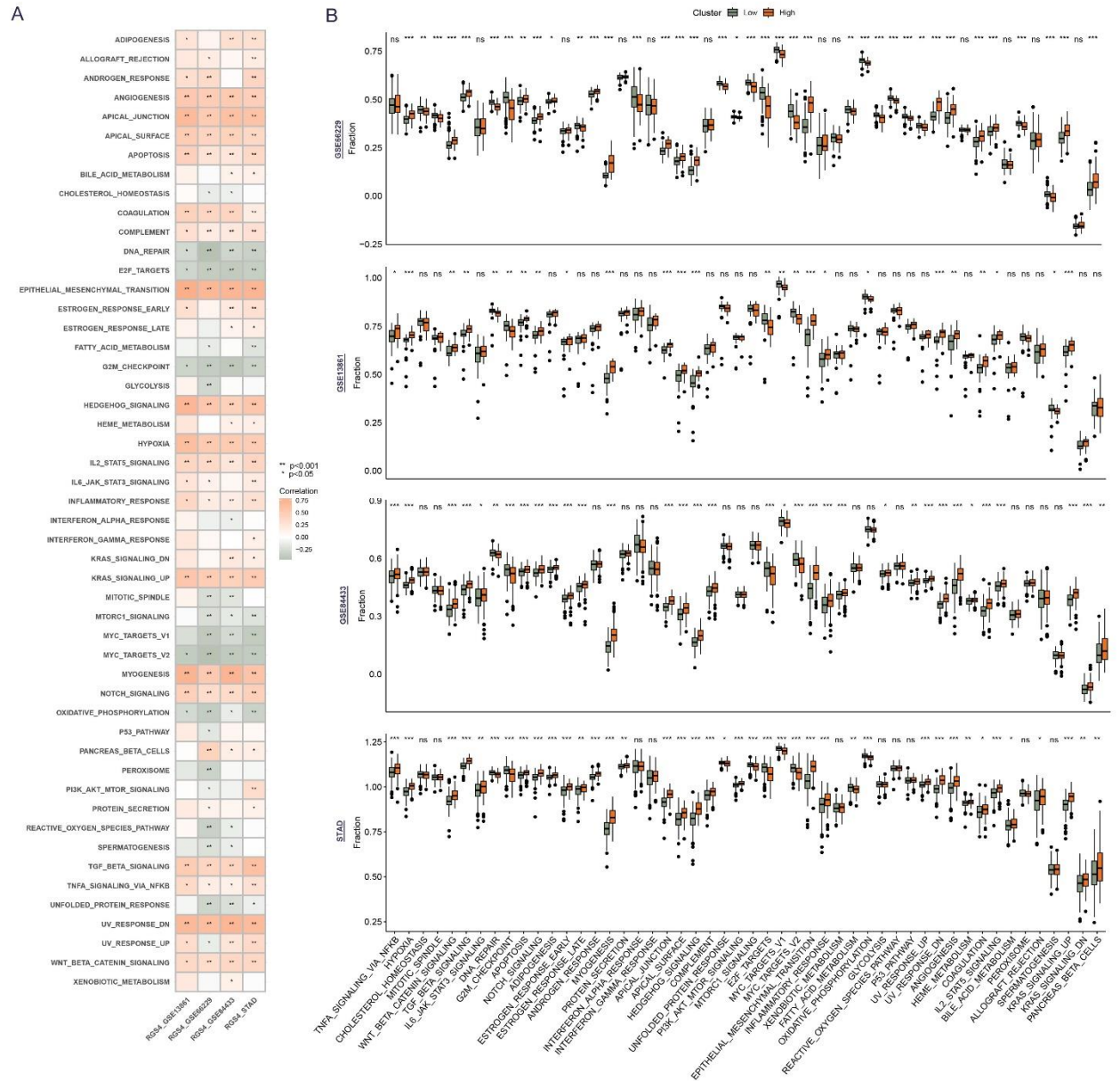

Supplement: Supplementary file 2 [file Image2.pdf]
