# Supplementary material for: Unraveling the prognostic significance of RGS gene family in gastric cancer and the potential implication of RGS4 in regulating tumor-infiltrating fibroblast
Source: Front Mol Biosci. 2024 Apr 17;11:1158852. doi: 10.3389/fmolb.2024.1158852 (PMC11061405; doi:10.3389/fmolb.2024.1158852)
Supplement: Supplementary file 3 [file Image3.pdf]

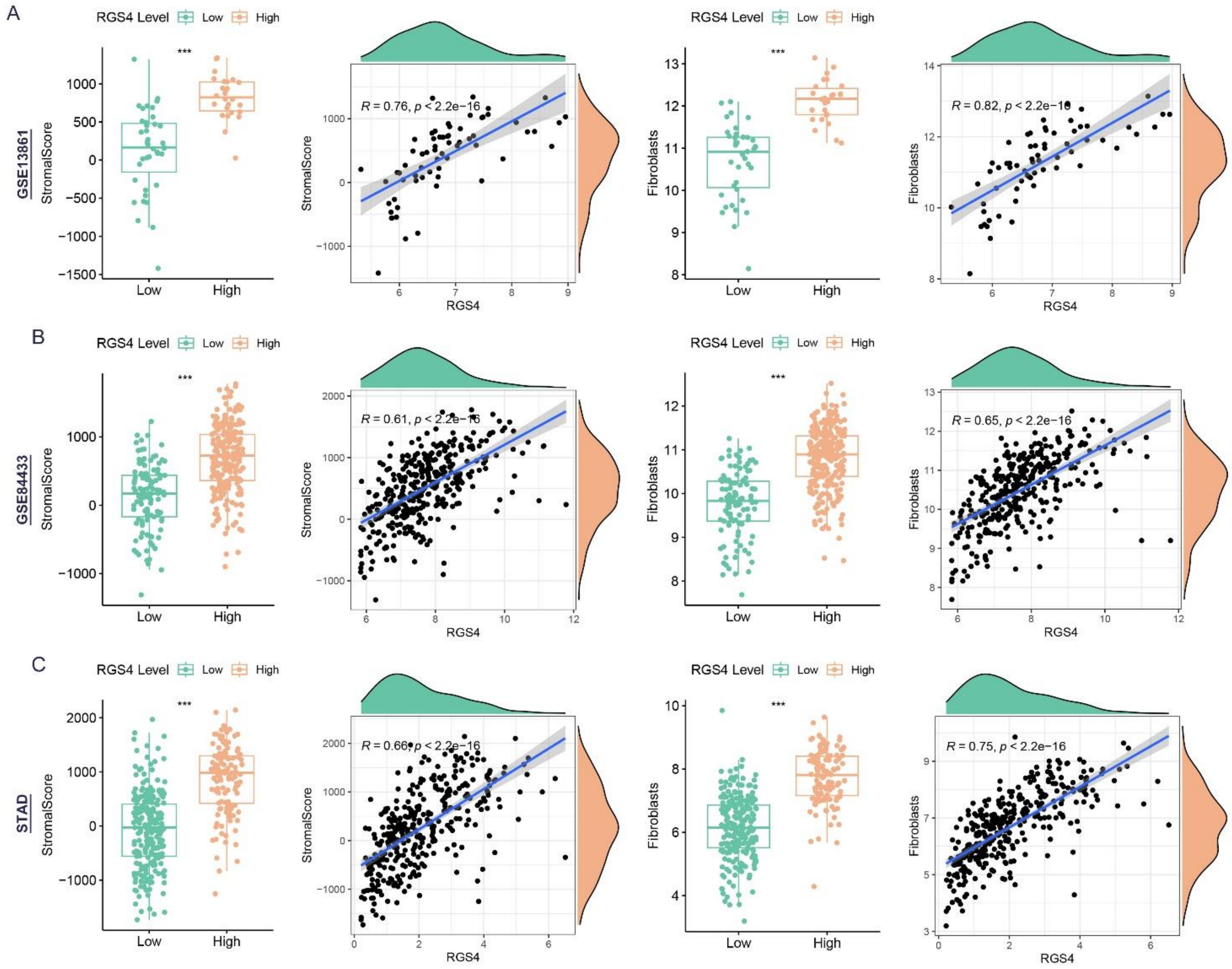

Supplementary Figure 3. Evaluation of the stromal score and the infiltration of fibroblasts of the RGS4 high- and low-group. (A) GSE13861. (B) GSE84433. (C) STAD.
