# Supplementary material for: Unraveling the prognostic significance of RGS gene family in gastric cancer and the potential implication of RGS4 in regulating tumor-infiltrating fibroblast
Source: Front Mol Biosci. 2024 Apr 17;11:1158852. doi: 10.3389/fmolb.2024.1158852 (PMC11061405; doi:10.3389/fmolb.2024.1158852)
Supplement: Supplementary file 4 [file Image1.pdf]

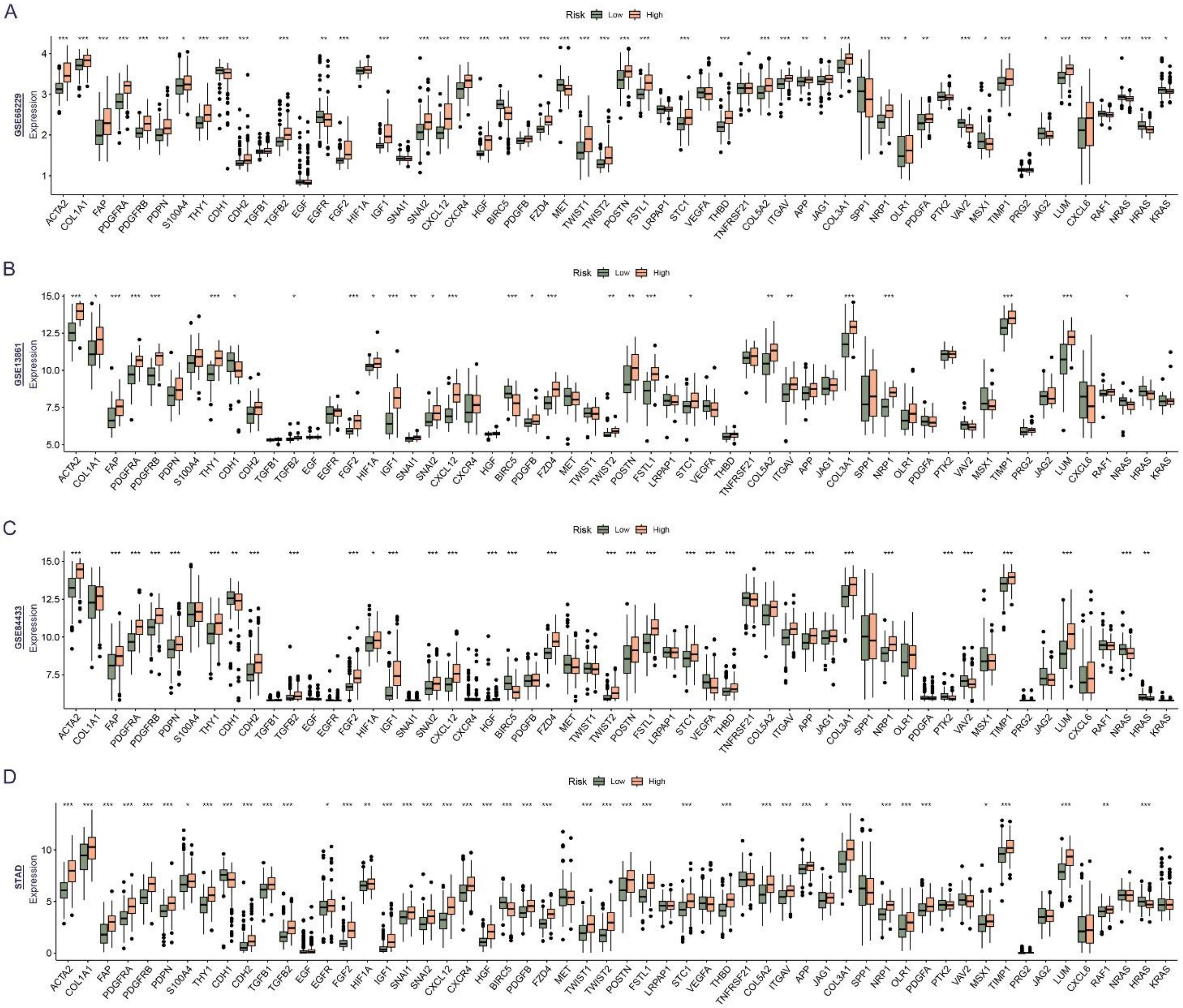

Supplementary Figure 1. Expression analysis of fibroblast, epithelial interstitial transformation, and angiogenesis-related genes in different risk groups in GSE66229 (A), GSE13861 (B), GSE84433 (C) and STAD (D).
